# SGLT2 Inhibitors in Elderly Patients: Clinical Perspectives from Metabolic and Cardiorenal Protection to Implementation

**DOI:** 10.3390/jcm15124578

**Published:** 2026-06-12

**Authors:** Iris Parrini, Roberto Ceravolo, Carmelo Massimiliano Rao, Fabiana Lucà, Michele Massimo Gulizia, Sandro Gelsomino, Nadia Ingianni, Giuseppe Carullo, Sebastiano Quartuccio, Stefania Renne, Claudio Bilato, Giovanna Geraci, Fabrizio Oliva, Federico Nardi, Massimo Grimaldi

**Affiliations:** 1 Cardiology Unit, Koelliker Hospital, 10134 Torino, Italy; irisparrini@libero.it; 2 Cardiology Unit, Giovanni Paolo II Hospital, 88046 Lamezia Terme, Italy; stefania.renne@virgilio.it; 3 Cardiology Unit, Santa Maria Degli Ungheresi Hospital, Polistena, 89024 Reggio Calabria, Italy; massimo.rao@libero.it (C.M.R.); sebastiano.quartuccio@gmail.com (S.Q.); 4 Cardiology Department, Grande Ospedale Metropolitano of Reggio Calabria, 89124 Reggio Calabria, Italy; fabiana.luca92@gmail.com; 5 Cardiology Department, Garibaldi Nesima Hospital, 95122 Catania, Italy; michele.gulizia60@gmail.com; 6 Cardiothoracic Department, Maastricht University Hospital, 6229 Maastricht, The Netherlands; sandro.gelsomino@gmail.com; 7 Cardiologic District, Azienda Sanitaria Provinciale Trapani, 91016 Trapani, Italy; nadiaing@hotmail.it; 8 Cardiology Unit, N. Giannettasio Hospital, 87064 Rossano, Italy; giuseppe.carul@tiscali.it; 9 Division of Cardiology, West Vicenza Hospitals, 36071 Arzignano, Italy; claudio.bilato@aulss8.veneto.it; 10 Cardiology Department, Sant’Antonio Abate Hospital, Erice, 91016 Trapani, Italy; giovannageraci@hotmail.com; 11 Cardiology Unit, ASST Grande Ospedale Metropolitano Niguarda, 20162 Milan, Italy; fabrizio.oliva@ospedaleniguarda.it; 12 Cardiology Department, Santo Spirito Hospital, Casale Monferrato, 15033 Alessandria, Italy; federico.nardi1@gmail.com; 13 Cardiology Department, General Regional Hospital “F. Miulli”, Acquaviva Delle Fonti, 70021 Bari, Italy; m.grimaldi@miulli.it

**Keywords:** SGLT2 inhibitors, elderly, frailty, heart failure, chronic kidney disease, cardiorenal protection

## Abstract

The prevalence of diabetes and heart failure rises sharply with age, and their coexistence amplifies cardiovascular and renal risk. Elderly patients display unique clinical and biological profiles characterised by frailty, multimorbidity, and pharmacodynamic variability that challenge conventional treatment strategies. Sodium–glucose co-transporter-2 inhibitors (SGLT2i) have emerged as a cornerstone of cardio–renal–metabolic protection, with the most consistent cardiovascular benefit being the reduction in heart failure hospitalisation, whereas effects on cardiovascular death and major adverse cardiovascular events vary according to baseline cardiovascular risk, heart failure phenotype, diabetic status, and trial design. However, real-world use among the elderly remains limited due to concerns about tolerability, polypharmacy, and cost. This review analyses the pharmacological rationale and evidence base for SGLT2i therapy in older adults, highlighting mechanisms beyond glucose control, quantitative data from pivotal trials, and practical issues for geriatric implementation.

## 1. Introduction

Population ageing has transformed the epidemiology of cardiometabolic disease. More than one-third of individuals with type 2 diabetes mellitus (T2DM) in Western countries are ≥65 years old, and the incidence of heart failure (HF) and chronic kidney disease (CKD) increases exponentially with age [1]. Older adults also accumulate multiple risk factors, including arterial hypertension (AH), obesity, sarcopenia, and endothelial dysfunction, which potentiate atherothrombotic and microvascular damage [2]. Managing these patients requires therapies that are not only effective but also compatible with frailty, multimorbidity, functional vulnerability, renal impairment, polypharmacy, and heterogeneous life expectancy. Traditional glucose-lowering strategies were largely glycemic-centric, and their cardiovascular (CV) neutrality or potential harm in frail elders limited their use [3]. The advent of SGLT2 inhibitors (SGLT2i) has redefined this paradigm by demonstrating consistent cardiorenal benefits independent of glycemic control [4,5,6]. However, these benefits are not homogeneous across CV endpoints. The reduction in heart failure hospitalisation (HHF) is the most reproducible finding, while effects on CV death and major adverse CV events (MACE) are more context-dependent and differ between atherosclerotic cardiovascular disease (ASCVD), HF with reduced Ejection Fraction (HFrEF), HF with preserved Ejection Fraction (HFpEF), CKD, diabetic and non-diabetic populations Large outcome trials, EMPA-REG OUTCOME, CANVAS, DECLARE-TIMI 58, DAPA-HF, EMPEROR-Reduced and EMPEROR-Preserved, have shown relative risk reductions of 25–35% in heart-failure hospitalisation and slower renal decline across diabetic and non-diabetic populations [7,8,9,10]. Subgroup analyses suggest preserved efficacy in participants aged ≥ 75 years, although these individuals remain underrepresented in randomised studies and are not necessarily representative of frail, dependent, cognitively impaired, or institutionalised older adults. Importantly, recent meta-analyses specifically focused on elderly populations have reinforced this evidence. In pooled analyses including patients aged ≥ 65 years, SGLT2 inhibitors were associated with significant reductions in HHF and renal outcomes, with consistent relative risk reductions across age groups, including those aged ≥ 75 years [11,12]. Nevertheless, most available analyses remain age-stratified rather than truly geriatric, as they rarely include systematic assessment of frailty, functional dependence, sarcopenia, cognitive impairment, falls, continence problems, caregiver support, or nursing home residence. Moreover, patients aged ≥ 80 years, those with advanced frailty, severe dependency, cognitive impairment, malnutrition, recurrent falls, poor oral intake, or nursing home residence remain markedly underrepresented in randomized clinical trials, limiting the generalisability of these findings to the most vulnerable geriatric populations. Accordingly, this review does not simply extrapolate evidence from age-defined subgroups, but applies a geriatric framework to SGLT2 inhibitor therapy, integrating efficacy, safety, frailty assessment, functional status, sarcopenia, orthostatic vulnerability, dehydration risk, cognitive impairment, caregiver involvement, shared decision-making, and situations in which treatment initiation may be inappropriate. In geriatric practice, treatment goals extend beyond HbA1c reduction to include maintaining functional independence, preventing hospitalisations, preserving quality of life, avoiding treatment-related functional decline, and aligning therapy with patient-centred priorities. Therefore, the integration of SGLT2 inhibitors into therapeutic algorithms for older adults requires careful evaluation of physiological changes with ageing, polypharmacy, orthostatic tolerance, hydration status, nutritional reserve, continence issues, cognitive function, medication self-management, caregiver support, and the balance between cardiorenal benefits and potential adverse events, such as volume depletion or genitourinary infections [13].

In older adults, therapeutic decisions should not be based on chronological age alone. A frailty-oriented approach is more appropriate, as biological age, functional capacity, cognitive status, comorbidity burden, and social support often better reflect treatment tolerance and clinical priorities than age itself. From a practical perspective, older patients may be broadly classified as robust, pre-frail, or frail, and each category may require a different balance between expected cardiorenal benefit, tolerability, monitoring intensity, and goals of care. In this setting, the prescription of SGLT2 inhibitors may be better supported by a multidimensional geriatric assessment including functional status, mobility, cognition, nutritional status, polypharmacy, and risk of dehydration or falls.

## 2. Materials and Methods

### 2.1. Search Strategy

This systematic review was conducted in accordance with the Preferred Reporting Items for Systematic Reviews and Meta-Analyses (PRISMA) statement [14]. The search strategy was defined by two investigators and subsequently reviewed by the senior authors to ensure methodological consistency. A comprehensive literature search was performed to identify studies evaluating the efficacy and safety of sodium–glucose co-transporter 2 (SGLT2) inhibitors in older adults. The following electronic databases were systematically searched: PubMed/MEDLINE. The search strategy combined Medical Subject Headings (MeSH) and free-text terms, including: “SGLT2 inhibitors”, “sodium–glucose co-transporter 2”, “elderly”, “older adults”, “ageing”, “frailty”, “heart failure”, “cardiovascular outcomes”, “renal outcomes”, and “chronic kidney disease”. The search was complemented by manual screening of the reference lists of relevant original articles and reviews to identify additional eligible studies. No predefined time limits were applied. Only studies published in English were considered.

### 2.2. Selection Criteria

Studies were selected according to predefined inclusion and exclusion criteria. Inclusion criteria were: (i) studies enrolling adult patients with T2DM, HF, or CKD; (ii) studies including older populations (generally defined as ≥65 or ≥75 years) or reporting age-stratified analyses; (iii) studies evaluating treatment with SGLT2 inhibitors (e.g., empagliflozin, dapagliflozin, canagliflozin, or related agents); (iv) studies reporting cardiovascular, HF or renal outcomes; (v) randomised controlled trials, post hoc analyses, or meta-analyses. Exclusion criteria were: (i) studies without age-specific or age-stratified data; (ii) studies not reporting relevant cardiovascular, renal, or HF outcomes; (iii) non-original articles (e.g., narrative reviews, editorials, letters); (iv) preclinical or animal studies.

### 2.3. Outcomes and Definitions

The primary outcomes of interest were: (i) MACE, defined as a composite of CV death, myocardial infarction, or stroke; (ii) HHF; (iii) progression of kidney disease, including decline in estimated glomerular filtration rate (eGFR) or development of end-stage kidney disease. Secondary outcomes included: (i) all-cause mortality; (ii) CV mortality; (iii) safety endpoints, particularly those relevant to older and frail populations, such as volume depletion, renal adverse events, and genitourinary infections. Given the heterogeneity across studies, outcome definitions were based on those reported in the original trials.

### 2.4. Study Selection and Data Extraction

The study selection process followed PRISMA recommendations. Titles and abstracts were initially screened to identify potentially relevant studies, followed by full-text evaluation for eligibility. A total of 2488 records were identified through database searching. After screening, 2441 records were excluded. Overall, 47 full-text articles were assessed for eligibility, and 37 were excluded due to a lack of age-stratified data, insufficient reporting of outcomes, or an ineligible study design. A total of 10 studies met the inclusion criteria and were included in the qualitative synthesis. Data extraction was performed using a standardised approach. The following variables were collected: study design, population characteristics, sample size, age distribution, type of SGLT2 inhibitor, comparator, duration of follow-up, CV outcomes, HF outcomes, renal outcomes, and safety data.

### 2.5. Quality Assessment and Risk of Bias

Given the nature of the included evidence, which primarily consisted of large randomised controlled trials and their subgroup analyses, risk of bias was assessed qualitatively. The evaluation considered study design, sample size, consistency of findings across trials, and robustness of age-stratified analyses. Particular attention was paid to the limited representation of frail and very elderly populations, which may affect the external validity of the findings. Due to the heterogeneity in study design and outcome reporting, a formal quantitative risk-of-bias scoring system was not applied.

### 2.6. Statistical Analysis

Due to the heterogeneity in study design, populations, and reporting of age-specific outcomes, a quantitative meta-analysis was not performed. Instead, a qualitative synthesis of the evidence was conducted, focusing on consistency of treatment effects across age groups, clinical settings, and endpoints. The analysis aimed to identify patterns of benefit across cardiovascular, renal, and HF outcomes, with particular emphasis on elderly populations.

## 3. Results

### 3.1. Study Selection and Characteristics

**Figure 1:**
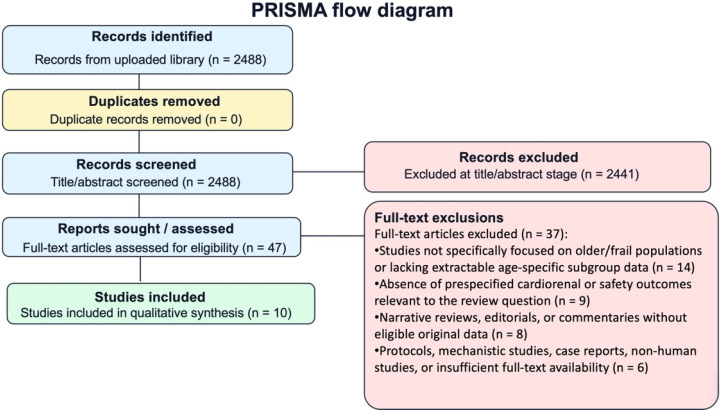
PRISMA flow diagram of study selection. The diagram illustrates the process of study identification, screening, eligibility assessment, and inclusion in this systematic review. A total of 2488 records were identified through database searching, with no duplicates removed. After title and abstract screening, 2441 records were excluded. Overall, 47 full-text articles were assessed for eligibility, of which 37 were excluded due to lack of age-specific data, absence of relevant CV or renal outcomes, non-original study design, or insufficient full-text availability. Ultimately, 10 studies were included in the qualitative synthesis.

The study selection process is summarised in the PRISMA flow diagram (Figure 1). A total of 2488 records were identified through database searching. After removal of non-relevant studies based on title and abstract screening, 47 full-text articles were assessed for eligibility. Of these, 37 were excluded due to lack of age-stratified analyses, insufficient reporting of outcomes, or ineligible study design.

Ultimately, 10 studies were included in the qualitative synthesis. These consisted of randomised controlled trials, subgroup analyses, and meta-analyses evaluating the effects of SGLT2 inhibitors in older populations. The included studies varied in population characteristics, with patients affected by T2DM, HF (both HFrEF and HFpEF), and CKD. The proportion of older participants differed across trials, and frailty was not systematically assessed in most studies.

### 3.2. Pharmacological Rationale and Mechanism of Action

**Figure 2:**
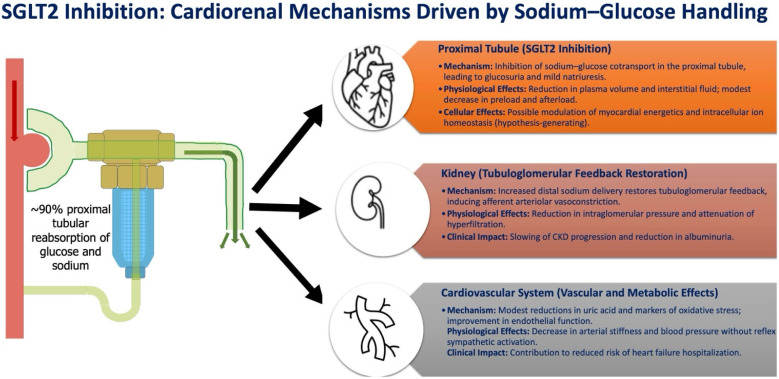
Cardiorenal and systemic mechanisms of SGLT2 inhibition. Sodium–glucose co-transporter-2 (SGLT2) inhibitors induce glycosuria and natriuresis, generating an “energy–sodium drain” that contributes to coordinated cardiovascular, renal, and vascular benefits. At the cardiac level, mild osmotic diuresis and reduced intracellular sodium, partly mediated by inhibition of the Na^+^/H^+^ exchanger, promote hemodynamic unloading and improve myocardial energetics by enhancing mitochondrial efficiency. In the kidney, restoration of tubuloglomerular feedback reduces intraglomerular pressure, attenuates hyperfiltration, and limits albuminuria, thereby protecting against progressive renal injury. Systemically, SGLT2 inhibition is associated with reductions in oxidative stress and uric acid levels, improved endothelial function, and modest decreases in arterial stiffness and blood pressure, contributing to overall CV and renal protection.

SGLT2i selectively blocks the sodium–glucose co-transporter-2 located in the proximal renal tubule, reducing reabsorption of approximately 90% of filtered glucose [15]. The resulting glycosuria promotes mild osmotic diuresis and natriuresis, leading to modest reductions in plasma volume, blood pressure, and body weight [16]. Each tablet thereby generates an “energy and sodium drain” that alleviates cardiac preload and afterload, lowers interstitial congestion, and indirectly improves ventricular efficiency (Figure 2).

Beyond these hemodynamic effects, SGLT2 inhibitors are thought to exert several metabolic and molecular actions that may be particularly relevant in the elderly myocardium. However, it should be acknowledged that the relative contribution of these mechanisms to clinical outcomes, especially in older and frail populations, remains incompletely defined. A shift in substrate utilisation from glucose to ketone bodies may increase the efficiency of ATP production per molecule of oxygen, potentially enhancing myocardial performance [17]. Moreover, inhibition of the Na^+^/H^+^ exchanger has been associated with reduced intracellular sodium and calcium overload, which may contribute to improved mitochondrial function and decreased oxidative stress [18]. In addition, restoration of tubuloglomerular feedback and consequent reduction in intraglomerular pressure are well-described mechanisms contributing to nephroprotection [19]. Furthermore, modest reductions in arterial stiffness and improvements in endothelial function have been observed, although the underlying pathways remain only partially understood [20]. Collectively, these mechanisms are biologically plausible and supported by a growing body of experimental evidence and may contribute to the observed clinical benefits of SGLT2 inhibitors across the cardiorenal continuum, although their relative contributions to outcomes, particularly in older and frail populations, remain incompletely defined. However, direct causal links, particularly in elderly populations, have not been fully established and should be interpreted with caution. A schematic overview of the proposed mechanisms and their potential clinical implications is summarised in Table 1. Pharmacokinetic properties are generally favourable in advanced age: SGLT2 inhibitors are orally bioavailable and undergo hepatic glucuronidation; however, thresholds for initiation and continuation may vary across individual agents according to renal function, approved indications, and prescribing recommendations [21,22]. However, decreased renal glucose filtration with ageing may attenuate glycemic efficacy while maintaining CV benefits, an aspect important for patient counselling. From a geriatric viewpoint, the absence of significant hypoglycaemia, weight gain, or sympathetic activation distinguishes SGLT2i from sulfonylureas or insulin. This safety advantage underpins their recommendation as first-line or add-on therapy in older adults with T2DM and high CV risk [22].

### 3.3. Pathophysiological Context of Ageing and SGLT2 Modulation

Ageing is accompanied by progressive cardiovascular, renal, and metabolic changes that may influence the clinical context in which SGLT2 inhibitors exert their effects. However, these age-related alterations are heterogeneous and do not develop uniformly across all older adults. Arterial stiffening, left ventricular hypertrophy, impaired diastolic relaxation, and nephron loss may contribute to the development of HFpEF and CKD, but their extent and clinical expression vary substantially according to biological ageing, comorbidity burden, and cumulative exposure to CV risk factors [23]. Endothelial dysfunction, chronic low-grade inflammation (“inflammaging”), and mitochondrial decline further reduce nitric oxide bioavailability and energy efficiency [24]. These processes lead to increased oxidative stress and vascular resistance, diminishing the heart–kidney axis adaptability to hemodynamic stressors.

In the kidney, ageing is associated with reduced renal plasma flow, glomerulosclerosis, and diminished tubular reabsorption reserve. Experimental and disease-specific evidence suggests that SGLT2 expression and activity may be increased in diabetic kidneys, potentially contributing to enhanced glucose and sodium reabsorption; however, similar upregulation in senescent nephrons has not been consistently demonstrated in human studies and should therefore be interpreted with caution [25]. By blocking SGLT2, these drugs may counter maladaptive hyperfiltration, lower intraglomerular pressure, and reduce proximal tubular oxygen demand, mechanisms that are thought to contribute to nephroprotection, including in older patients [26].

The hemodynamic unloading produced by SGLT2 inhibition may be particularly relevant in older patients with HF, especially in the presence of congestion, although tolerance to preload reduction is not uniform and depends on clinical factors such as frailty status, HF phenotype, autonomic function, and baseline volume status The mild and sustained diuretic effect of SGLT2 inhibitors may provide a smoother decongestive strategy than loop diuretics, with less pronounced neurohormonal activation overall, although transient activation of the renin–angiotensin–aldosterone system may occur early after treatment initiation [27]. This contributes to the lower rates of HF hospitalisation observed in trials.

At the vascular level, SGLT2i mitigate endothelial dysfunction by reducing uric acid, advanced glycation end products, and markers of oxidative stress [28]. Improvement in arterial compliance may contribute to better vascular function, whereas the potential role of endothelial progenitor cell mobilisation in enhancing microvascular perfusion remains speculative and has not been consistently demonstrated, particularly in older populations. Such effects are independent of glycemic status, reinforcing their role in non-diabetic patients with HF.

In summary, the pathophysiological context of ageing provides a rationale for considering SGLT2 inhibition in selected older adults, particularly when HF, CKD, or congestion are present. However, the extent to which the proposed effects on oxidative stress, endothelial dysfunction, mitochondrial metabolism, and “cardiorenal ageing” directly translate into clinical benefit in very old or frail populations remains uncertain. Therefore, mechanistic hypotheses should be clearly distinguished from outcomes demonstrated in randomised trials. In summary, the pathophysiological context of ageing provides a rationale for considering SGLT2 inhibition in selected older adults, particularly when HF, chronic kidney disease, or congestion are present. However, the extent to which the proposed effects on oxidative stress, endothelial dysfunction, mitochondrial metabolism, and “cardiorenal ageing” directly translate into clinical benefit in very old or frail populations remains uncertain. Therefore, mechanistic hypotheses should be clearly distinguished from outcomes demonstrated in randomized trials.

### 3.4. Clinical Efficacy of SGLT2 Inhibitors in the Elderly

#### 3.4.1. Cardiovascular Outcomes: Endpoint- and Phenotype-Specific Evidence

**(a) MACE and cardiovascular death in T2DM/ASCVD trials**

The CV effects of SGLT2 inhibitors extend beyond glycemic control and are preserved in older adults; however, these effects differ substantially across clinical endpoints and patient populations. Indeed, the CV effects of SGLT2 inhibitors should not be interpreted as a single homogeneous “CV protection” effect. Across trials, the most consistent and reproducible benefit is the reduction in HHF, whereas effects on CV death and major adverse CV events are more variable and depend on the population studied, baseline CV risk, HF phenotype, and trial design. In the landmark EMPA-REG OUTCOME trial, empagliflozin reduced the composite of CV death, non-fatal myocardial infarction, or stroke by 14% (HR 0.86; 95% CI 0.74–0.99) and CV mortality by 38% [7]. Sub-analyses confirmed similar effect sizes in participants aged > 75 years, with an absolute risk reduction almost doubling due to higher baseline event rates. Importantly, the reduction in HHF observed in EMPA-REG OUTCOME represents one of the most consistent findings across the SGLT2 inhibitor class, whereas the impact on MACE appears more dependent on baseline atherosclerotic risk. However, these findings should not be automatically extrapolated to all elderly patients with HF or CKD, particularly those without T2DM or without established ASCVD. The CANVAS programme (canagliflozin) yielded a 33% decrease in HF hospitalisation (HR 0.67; 95% CI 0.52–0.87) and significant renal protection [8]. Older individuals (mean age ≈ 63 years; upper quartile ≥ 70 years) exhibited parallel benefits without increased fracture or amputation rates after protocol adjustments. DECLARE-TIMI 58 (dapagliflozin) enrolled >17,000 patients (41% ≥ 65 yrs) and confirmed a 27% reduction in HF hospitalisation (HR 0.73; 95% CI 0.61–0.88) with neutral MACE effects [9]. This reinforces the concept that SGLT2 inhibitors should not be expected to uniformly reduce atherosclerotic events, but rather to consistently impact HF-related outcomes across different populations. In contrast to EMPA-REG OUTCOME, DECLARE-TIMI 58 showed a neutral effect on MACE while demonstrating a significant reduction in HHF. This distinction is clinically important in older adults because the expected benefit may be predominantly related to the prevention of HF events rather than the prevention of myocardial infarction or stroke. These findings indicate that SGLT2 inhibitors should not be expected to provide uniform protection across all CV outcomes, but rather to exert endpoint-specific benefits that vary with baseline risk and clinical context.

**(b) Heart failure outcomes in HFrEF**

In DAPA-HF and EMPEROR-Reduced, which included both diabetic and non-diabetic patients with HFrEF patients (mean age ≈ 67 years), dapagliflozin and empagliflozin, respectively, lowered the primary composite of CV death or HF hospitalisation by 26% (HR 0.74; 95% CI 0.65–0.85) and 25% (HR 0.75; 95% CI 0.65–0.86) [10,13]. The magnitude of benefit was uniform across age groups up to 85 years. In HFrEF, therefore, the evidence is robust and consistent, with SGLT2 inhibitors reducing the composite of worsening HF or CV death irrespective of diabetic status. This supports their role as disease-modifying therapies in HFrEF, where the reduction in the composite endpoint is driven by both decreased HF events and, to a lesser extent, CV mortality. In this setting, the benefit reflects both reduction in HF events and, to a lesser extent, CV mortality, supporting a disease-modifying effect.

**(c) Heart failure outcomes in HFpEF**

**Figure 3:**
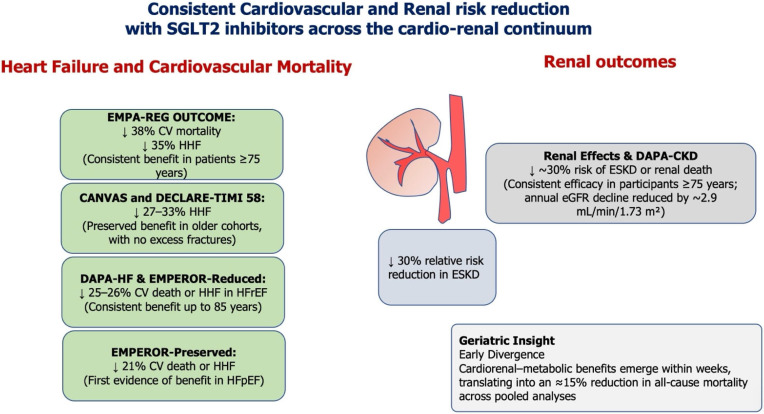
Endpoint-specific cardiovascular and renal effects of SGLT2 inhibitors across the cardiorenal continuum. The symbol “↓” indicates a reduction/decrease.

For HFpEF, predominant in older adults, *EMPEROR-Preserved* and *DELIVER* demonstrated, for the first time, a statistically significant 21% reduction in the composite endpoint (HR 0.79; 95% CI 0.69–0.90), irrespective of diabetic status [15,16]. In HFpEF, a phenotype particularly common in older adults, the benefit appears to be driven mainly by a reduction in worsening HF events and HHF, whereas a consistent reduction in CV mortality has not been clearly demonstrated. This distinction is particularly relevant in older adults, in whom HFpEF is highly prevalent and clinical benefit is more likely to be derived from reduction in congestion and hospitalisation rather than prevention of atherosclerotic events. To avoid merging heterogeneous CV outcomes into a single protective narrative, Table 2 now distinguishes primary endpoints, key secondary endpoints, and the outcomes with the most robust evidence in older populations. Overall, the trial evidence indicates that the most consistent CV signal across SGLT2 inhibitor studies is the reduction in HHF or worsening HF. Effects on CV death are more evident in selected high-risk populations, whereas MACE reduction is not uniform across trials. In older adults, this distinction is clinically relevant because absolute benefit may be greater due to higher baseline risk, but tolerability depends on frailty, blood pressure reserve, renal function, congestion status, and concomitant therapies. Their hemodynamic, metabolic, and endothelial effects directly target mechanisms of diastolic dysfunction and microvascular inflammation, which are typical of senescent myocardium (Figure 3). In clinical practice, this implies that the expected benefit in older HFpEF patients is primarily related to symptom control and prevention of decompensation rather than reduction of atherosclerotic events.

**(d) Interpretation in older adults**

Overall, the available evidence indicates that the most robust and consistent CV effect of SGLT2 inhibitors is the reduction in HHF, while effects on CV death and MACE are more variable and context-dependent. In older adults, this distinction is essential for appropriate clinical interpretation and therapeutic decision-making. These effects should be interpreted in light of specific clinical endpoints, as SGLT2 inhibitors do not exert a uniform impact across all CV outcomes. Therefore, in older adults, therapeutic expectations should be aligned with the predominant effect on HF outcomes rather than assuming a generalised reduction in all CV events. To provide additional clinical context on the magnitude of treatment benefit across guideline-directed medical therapies (GDMTs) in HF, approximate number needed to treat (NNT) values derived from landmark trials are summarised in Table 3.

**Table 2:** Endpoint-specific evidence from major SGLT2 inhibitor trials and relevance to older adults.

Trial	Population (n)	Age ≥ 65 yrs (%)	Primary Endpoint	Key Secondary Endpoints	Most Robust Treatment Signal	HR [95% CI]/*p*	Interpretation in Older Adults
**EMPA-REG OUTCOME [7]**	7020 T2DM + ASCVD	28%	3-point MACE	CV death, HHF	Strong reduction in CV death and HHF	0.86 [0.74–0.99]; *p* = 0.04	Benefit consistent ≥ 75 yrs; driven by high baseline risk; frailty not assessed
**CANVAS Program [8]**	10,142 T2DM	34%	3-point MACE	HHF, renal outcomes	Reduction in HHF and renal events; MACE reduction present but less dominant	0.67 [0.52–0.87]; *p* = 0.001	Preserved benefit in older subgroups; limited data in frail elderly
**DECLARE-TIMI 58 [9]**	17,160 T2DM	41%	MACE and CV death/HHF (co-primary)	HHF, renal composite	Reduction in HHF; neutral effect on MACE	0.73 [0.61–0.88]; *p* < 0.001	Consistent HHF benefit ≥ 75 yrs; limited representation of very old patients
**DAPA-HF [10]**	4744 HFrEF ± DM	55%	Worsening HF or CV death	HHF, symptoms, renal outcomes	Robust reduction in HFrEF outcomes irrespective of diabetes	0.74 [0.65–0.85]; *p* < 0.001	Benefit consistent across age groups; selected population; frailty not systematically assessed
**EMPEROR-Reduced [13]**	3730 HFrEF	~50%	CV death or HHF	Total HHF, renal decline	Reduction in HF events with consistent effect across subgroups	0.75 [0.65–0.86]; *p* < 0.001	Consistent benefit in older adults; limited frailty characterization
**EMPEROR-Preserved [29]**	5988 HFpEF	45%	CV death or worsening HF	HHF, QoL, renal outcomes	Benefit mainly driven by reduction in HF events	0.79 [0.69–0.90]; *p* < 0.001	Highly relevant to the elderly; caution in frail or preload-dependent patients
**DAPA-CKD [30]**	4304 CKD ± DM	43%	Renal composite (ESKD, eGFR decline, renal death)	CV death, HHF	Strong renal protection and reduction in HF events	0.70 [0.59–0.82]; *p* < 0.001	Benefit maintained ≥ 75 yrs; limited evidence in advanced frailty

Abbr.: T2DM: type 2 diabetes mellitus; ASCVD: atherosclerotic cardiovascular disease; CV: cardiovascular; HHF: hospitalisation for heart failure; MACE: major adverse cardiovascular events; HFrEF: heart failure with reduced ejection fraction; HFpEF: heart failure with preserved ejection fraction; DM: diabetes mellitus; CKD: chronic kidney disease; ESKD: end-stage kidney disease; HR: hazard ratio; CI: confidence interval.

**Table 3:** Approximate number needed to treat (NNT) across guideline-directed medical therapies in heart failure.

Therapy	Landmark Trial	Population	Primary Outcome	Follow-Up	Approximate NNT
**SGLT2 inhibitors (dapagliflozin)**	DAPA-HF [10]	HFrEF (±diabetes)	CV death or HF hospitalisation	18 months	~21
**SGLT2 inhibitors (empagliflozin)**	EMPEROR-Reduced [13]	HFrEF (±diabetes)	CV death or HF hospitalisation	16 months	~19
**ARNI (sacubitril/valsartan)**	PARADIGM-HF [31]	HFrEF	CV death or HF hospitalisation	27 months	~21
**Beta-blockers (metoprolol)**	MERIT-HF [32]	HFrEF	All-cause mortality	12 months	~27
**Mineralocorticoid receptor antagonists (spironolactone)**	RALES [33]	HFrEF	All-cause mortality	24 months	~9
**ACE inhibitors (enalapril)**	SOLVD-Treatment [34]	HFrEF	All-cause mortality	41 months	~22

Abbreviations: ACE, angiotensin-converting enzyme; ARNI, angiotensin receptor–neprilysin inhibitor; CV, cardiovascular; HF, heart failure; HFrEF, heart failure with reduced ejection fraction; NNT, number needed to treat. NNT values are derived from individual landmark trials and are presented for contextual purposes only. Direct comparisons across therapies should be interpreted with caution due to differences in study populations, baseline risk, endpoints, and follow-up duration.

Major randomised clinical trials have consistently demonstrated that sodium–glucose co-transporter-2 (SGLT2) inhibitors reduce CV and renal events across a wide spectrum of cardiometabolic conditions. In patients with T2DM and established CVD, the EMPA-REG OUTCOME trial showed significant reductions in CV mortality and HHF. Similar reductions in HF events were observed in CANVAS and DECLARE-TIMI 58. In patients with HF, both with reduced and preserved ejection fraction, trials such as DAPA-HF, EMPEROR-Reduced, and EMPEROR-Preserved demonstrated meaningful reductions in CV death and HHF. In the renal setting, studies including CREDENCE and DAPA-CKD [30] reported approximately a 30% relative risk reduction in kidney failure or renal death, along with a slower decline in estimated glomerular filtration rate. Notably, these benefits emerge early after treatment initiation and have been observed across age subgroups. However, evidence in very old, frail, underweight, cognitively impaired, or nursing home populations remains limited, and much of the available evidence in these groups derives from subgroup, post hoc, or observational analyses.

Evidence specifically addressing older populations further supports these findings. A recent meta-analysis demonstrated that SGLT2 inhibitors significantly reduce the risk of HHF in patients aged ≥ 65 years, with no evidence of attenuation of the treatment effect compared with younger individuals [11]. These results are consistent with real-world data, which show comparable reductions in CV events and mortality among older patients treated with SGLT2 inhibitors in routine clinical practice [35]. Nevertheless, data in very elderly patients (≥80 years) remains limited. Although subgroup analyses suggest preserved efficacy, the small number of participants and potential selection bias preclude definitive conclusions in this population [36].

#### 3.4.2. Renal Outcomes

Renal outcome trials, CREDENCE, DAPA-CKD, and EMPA-Kidney, solidified SGLT2i as the first pharmacologic class after RAAS blockers to confer substantial nephroprotection. CREDENCE showed a 30% relative risk reduction in the composite of end-stage kidney disease, doubling of serum creatinine, or renal death (HR 0.70; 95% CI 0.59–0.82) [17]. DAPA-CKD extends these benefits to non-diabetic CKD, with consistent results in participants aged ≥ 75 years [18,30]. The estimated GFR declined by 2.9 mL/min/1.73 m^2^ compared with placebo.

However, in older adults, particularly those with CKD stage 4, this finding requires careful clinical interpretation. Progressive creatinine rise, symptomatic hypotension, dehydration, poor oral intake, acute infection, excessive diuresis, or concomitant treatment with loop or thiazide diuretics should prompt reassessment of volume status, temporary treatment interruption, or adjustment of concomitant therapy. Long-term trajectories demonstrate preserved renal function and fewer episodes of acute kidney injury compared with other diuretics [19].

#### 3.4.3. Metabolic and Systemic Effects

SGLT2 inhibition yields modest HbA1c reductions (~0.6%), 2–3 kg weight loss, and 4–5 mm Hg systolic BP lowering [20]. The early decline in eGFR observed after SGLT2 inhibitor initiation usually reflects hemodynamic resetting due to reduced intraglomerular pressure rather than structural kidney injury. In clinically stable patients, a small, non-progressive eGFR dip may be monitored without discontinuing treatment. However, in older adults, particularly those with CKD stage 4, this finding requires careful clinical interpretation. Progressive rise in creatinine, symptomatic hypotension, dehydration, poor oral intake, acute infection, excessive diuresis, or concomitant treatment with loop or thiazide diuretics should prompt reassessment of volume status, temporary treatment interruption, or adjustment of concomitant therapy. Initiation may be inappropriate or should be deferred in frail elderly patients with active volume depletion, recurrent dehydration, severe orthostatic symptoms, poor nutritional or fluid intake, acute illness, active genitourinary infection, or unstable renal function. Particular caution is also required in patients with recurrent urinary or genital infections, indwelling urinary catheters, urinary retention, severe incontinence, or poor self-care capacity, because these conditions may increase the clinical impact of genitourinary adverse events. In very old patients with advanced CKD, evidence remains less robust than in younger or less frail trial populations, and treatment decisions should therefore be individualised, ideally within a multidisciplinary cardiorenal or geriatric framework.

These effects, while modest, may contribute to symptom relief and global risk reduction in selected older adults, particularly by reducing congestion, dyspnoea, and blood pressure variability. However, in undernourished, sarcopenic, or frail older adults, even modest weight loss may be clinically relevant and potentially detrimental. In these patients, careful monitoring of nutritional status, body weight, muscle strength, and functional performance is recommended, and treatment decisions should balance cardiorenal benefits against the risk of further functional decline [21].

The cumulative cardio–renal–metabolic benefit translates into a ~15% reduction in all-cause mortality across pooled analyses [22]. Importantly, the benefit manifests early, within weeks, suggesting mechanisms beyond glycaemia.

### 3.5. Safety and Tolerability in Older Adults

#### 3.5.1. General Safety Profile

Across major trials, adverse event rates or rates of adverse events with SGLT2i were comparable to those with placebo, irrespective of age. Comparative analyses between elderly and younger populations indicate that, while the glucose-lowering effect of SGLT2 inhibitors may be slightly attenuated and the incidence of some adverse events modestly increased in older individuals, the overall safety profile remains favourable and consistent across age groups [12]. Real-world studies in elderly cohorts confirm these findings, showing good tolerability even in patients of advanced age, although closer clinical monitoring is warranted in more vulnerable individuals [37]. Discontinuation due to adverse effects occurred in ≈8–10% of subjects, slightly lower than with diuretics or GLP-1 receptor agonists [23]. The risk of hypoglycaemia remains minimal unless combined with insulin or sulfonylureas. In older adults with T2DM, initiation of SGLT2 inhibitors should prompt a careful reassessment of concomitant glucose-lowering therapies. In particular, dose reduction or deintensification of insulin or sulfonylureas may be appropriate to minimise the risk of hypoglycaemia, especially in frail individuals, those with reduced oral intake, or those with variable glycemic control. Glycemic targets should be individualised according to frailty status, comorbidity burden, life expectancy, and hypoglycaemia risk, rather than strictly pursuing conventional HbA1c thresholds. In this context, SGLT2 inhibitors are often part of a broader strategy of treatment simplification and risk reduction.

Volume depletion may lead to symptomatic hypotension, particularly in patients aged ≥ 80 years, in those receiving high-dose diuretics, and in frail individuals with reduced physiological reserve. In pooled analyses, orthostatic hypotension occurred in 3–5% of patients, compared with 2–4% on placebo [24]. This issue is especially relevant in older adults, in whom orthostatic symptoms may increase the risk of falls, functional decline, and treatment discontinuation. Gradual introduction of treatment, reassessment of concomitant diuretic therapy, and close monitoring of blood pressure, body weight, and orthostatic symptoms may mitigate this risk [24]. Dehydration deserves particular attention in frail elderly patients, especially in the setting of poor oral intake, intercurrent illness, cognitive impairment, or concomitant use of loop diuretics. In such patients, temporary treatment interruption and reinforcement of hydration advice are appropriate preventive measures. Euglycemic ketoacidosis remains a rare adverse event in T2DM, but clinicians should remain vigilant in older adults. The risk may increase in situations involving prolonged fasting, acute illness, perioperative periods, low carbohydrate intake, or significant metabolic stress. In older individuals, these conditions may be more frequent and less easily recognised, requiring proactive patient and caregiver education and, when appropriate, temporary treatment interruption [25].

#### 3.5.2. Renal Function and Electrolyte Balance

Elderly patients frequently have eGFR < 60 mL/min/1.73 m^2^. In older patients with reduced renal function, the use of SGLT2 inhibitors should be guided by the specific agent, approved eGFR thresholds, and clinical indication, as these are not uniform across the class [18]. This point is particularly relevant in geriatric patients, in whom renal function should not be interpreted only through a numerical eGFR threshold, but in the context of baseline kidney function, renal trajectory, volume status, frailty, comorbidities, nutritional reserve, concomitant diuretic therapy, and overall treatment goals. Importantly, glucose-lowering efficacy progressively declines as eGFR declines due to reduced filtered glucose load, whereas CV and renal protective effects may persist at lower eGFR values through hemodynamic and non-glycemic mechanisms. This distinction should be clearly discussed with patients and caregivers, as reduced glycemic efficacy at low eGFR should not be automatically interpreted as therapeutic failure when the indication is cardiorenal protection. The early decline in eGFR observed after SGLT2 inhibitor initiation usually reflects hemodynamic resetting due to reduced intraglomerular pressure rather than structural kidney injury. In clinically stable patients, a small, non-progressive eGFR dip may be monitored without discontinuing treatment. However, in older adults, particularly those with CKD stage 4, the same finding requires more careful clinical interpretation. Progressive rise in creatinine, symptomatic hypotension, dehydration, poor oral intake, acute infection, excessive diuresis, or concomitant treatment with loop or thiazide diuretics should prompt reassessment of volume status, temporary treatment interruption, or adjustment of concomitant therapy. Initiation may be inappropriate or should be deferred in frail elderly patients with active volume depletion, recurrent dehydration, severe orthostatic symptoms, poor nutritional or fluid intake, acute illness, active genitourinary infection, or unstable renal function. Particular caution is also required in patients with recurrent urinary or genital infections, indwelling urinary catheters, urinary retention, severe incontinence, or poor self-care capacity, because these conditions may increase the clinical impact of genitourinary adverse events. In very old patients with advanced CKD, evidence remains less robust than in younger or less frail trial populations, and treatment decisions should therefore be individualised, ideally within a multidisciplinary cardiorenal or geriatric framework. No consistent signal of excess hyperkalaemia or major electrolyte imbalance has been observed in pivotal trials; however, renal function, electrolytes, blood pressure, body weight, hydration status, and symptoms of volume depletion should be monitored closely after initiation and during acute clinical changes. Evidence in end-stage kidney disease and dialysis settings remains limited, and SGLT2 inhibitors should not be presented as routinely maintainable until dialysis in all older patients. Rather, continuation of advanced CKD should be reassessed periodically according to indication, tolerability, intercurrent illness, renal trajectory, hydration status, life expectancy, and patient-centred goals of care.

#### 3.5.3. Genitourinary and Other Adverse Events

Mycotic genital infections remain the most frequent adverse event (4–6% in women and approximately 2% in men) and generally respond to local therapy [27,28]. In older adults, preventive strategies such as genital hygiene, adequate hydration, and structured patient or caregiver education are particularly important. Urinary tract infections do not appear to increase substantially overall, although greater caution is warranted in patients with urinary incontinence, indwelling catheters, poor self-care, or advanced frailty, in whom infectious complications may be less well tolerated. Concerns about amputations and fractures seen in early canagliflozin studies were not replicated in subsequent trials or real-world analyses following protocol modification [28]. Importantly, a pooled meta-analysis, including >90,000 patients, showed no increase in malignancy or hepatic injury [38]. Particular caution is required in patients with poor hygiene, cognitive impairment, limited self-care capacity, or dependence on caregivers, as these factors may increase both the incidence and the clinical impact of genitourinary infections.

#### 3.5.4. Drug Interactions and Polypharmacy

The geriatric population frequently takes >5 drugs daily. SGLT2i show negligible CYP-mediated interactions; however, additive effects with loop or thiazide diuretics can enhance natriuresis. Temporary discontinuation during acute illness (“sick-day rules”) is particularly important in older adults and may help prevent dehydration, prerenal azotemia, and metabolic decompensation, especially in the presence of poor oral intake, vomiting, diarrhoea, or prolonged fasting [25,39]. Co-administration with ACEI/ARB/ARNI is generally safe and may even enhance nephroprotection [39]. Dose adjustment of other antihypertensives may be required as blood pressure improves.

#### 3.5.5. Quality of Life and Functional Status

Beyond numerical outcomes, SGLT2i significantly improves health-related quality of life (HRQoL). In DAPA-HF, KCCQ overall summary scores increased by 5.8 points compared with 3.5 with placebo, reflecting better physical limitation and symptom burden [10]. Similar findings were observed in DELIVER, particularly among women and those aged ≥ 70 years [16]. Enhanced functional capacity, reduced oedema, and fewer hospitalisations translate into preserved independence, which is a key goal of geriatric care [40].

### 3.6. Frailty and Geriatric Syndromes

#### 3.6.1. Frailty as a Therapeutic Determinant

Frailty, defined by reduced physiological reserve and impaired resilience to stressors, is highly prevalent among older patients with HF, T2DM, and CKD, and is frequently associated with sarcopenia, malnutrition, cognitive impairment, functional dependence, and polypharmacy [41,42]. In this context, therapeutic decisions should consider not only cardiorenal efficacy but also functional status, orthostatic tolerance, hydration reserve, nutritional vulnerability, medication self-management, and caregiver support.

**Figure 4:**
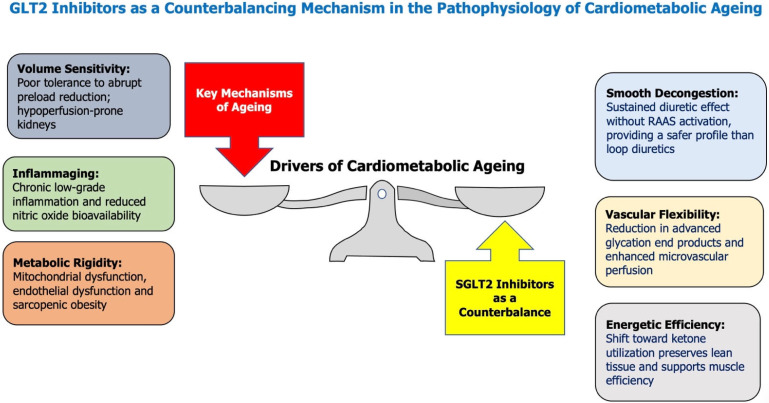
GLT2 inhibitors as a counterbalancing mechanism in cardiometabolic ageing.

From a practical standpoint, older adults may be broadly categorised as robust, pre-frail, or frail. Robust individuals are generally suitable for guideline-directed treatment strategies similar to those used in younger patients. Pre-frail individuals may still derive substantial benefit from SGLT2 inhibitors, but usually require closer surveillance of hydration status, orthostatic symptoms, renal function, and body weight. In frail patients, treatment decisions should be more individualised and integrated with prognosis, functional dependence, nutritional status, caregiver support, and overall goals of care. In this context, comprehensive geriatric assessment may help refine clinical decision-making by incorporating domains such as activities of daily living, gait and mobility, cognition, mood, nutrition, comorbidity burden, and medication review. Although no single geriatric assessment tool has been specifically validated to guide SGLT2 inhibitor prescription, routine geriatric instruments may improve patient selection and help identify those at greater risk of adverse events, dehydration, falls, or treatment discontinuation. SGLT2 inhibitors remain an attractive option in many older adults because of their oral administration and multidimensional cardiorenal benefits, but their use should be adapted to the individual frailty profile (Figure 4). Data specifically addressing frail populations remains limited. However, real-world evidence suggests that SGLT2 inhibitors can be used safely in older and potentially frail individuals, with beneficial effects on HF-related outcomes and clinical stability [12,36]. Notably, frailty has not been systematically assessed in randomised trials, and validated frailty indices have rarely been incorporated into study designs, representing a major gap in the current evidence base.

Observational data suggest that SGLT2 inhibitor initiation in frail patients with T2DM may be associated with lower all-cause mortality compared with DPP-4 inhibitors after propensity matching [29]. These findings are clinically relevant, but should be interpreted with caution because of the potential for residual confounding, selection bias, and differences in baseline functional status. Improved hemodynamics and congestion control may contribute to reduced fatigue and better exercise tolerance, although these functional benefits remain insufficiently characterised in dedicated geriatric studies. In the SOLD registry, frail individuals treated with SGLT2 inhibitors reported fewer HF readmissions and better KCCQ physical function scores [43]. In addition, data on functional outcomes, including mobility, risk of falls, and maintenance of independence, are currently lacking. Although improvements in HF status and congestion may indirectly translate into better functional capacity, these outcomes have not been formally evaluated in clinical trials of SGLT2 inhibitors in elderly populations [11]. Similarly, patients residing in nursing homes or with advanced frailty are largely excluded from randomised trials. As a result, the applicability of SGLT2 inhibitors in these populations remains uncertain and should be individualised, taking into account clinical complexity, comorbidities, and life expectancy [36].

Ageing is associated with several pathophysiological alterations that contribute to increased CV vulnerability, including chronic low-grade inflammation (“inflammaging”), mitochondrial dysfunction, endothelial impairment, metabolic rigidity, and heightened sensitivity to changes in intravascular volume. These processes promote frailty, reduced physiological reserve, and a higher burden of cardiometabolic disease in older adults. SGLT2i may counterbalance these mechanisms through multiple complementary effects. Their sustained osmotic diuresis promotes gradual and hemodynamically stable decongestion without significant activation of the renin–angiotensin–aldosterone system. In addition, metabolic reprogramming toward ketone utilisation improves energetic efficiency and may help preserve lean tissue and muscle function. At the vascular level, SGLT2 inhibition reduces oxidative stress and advanced glycation end products while improving microvascular perfusion. Together, these mechanisms may contribute to clinically relevant benefits, including improved congestion control, reduced fatigue and oedema, better quality of life, and lower risk of hospitalisation. However, in frail or highly vulnerable older adults, these potential benefits should be balanced against individual risks, including dehydration, orthostatic symptoms, weight loss, genitourinary complications, and treatment burden.

#### 3.6.2. Sarcopenia and Body Composition

Elderly patients with T2DM frequently exhibit sarcopenic obesity, characterised by reduced muscle mass in the presence of excess visceral adiposity. The effect of SGLT2 inhibitors on muscle mass and sarcopenia risk remains a matter of debate. Available data suggest that the modest weight loss associated with these agents is predominantly driven by fat mass reduction, with relative preservation of lean tissue in most patients [44]. However, in very old, frail, malnourished, or underweight individuals, excessive weight loss or reduced caloric intake could theoretically contribute to worsening sarcopenia and functional decline. For this reason, periodic assessment of body weight, nutritional status, muscle strength, and physical performance should be considered in vulnerable elderly patients, and exercise and nutritional counselling remain essential adjuncts.

#### 3.6.3. Cognitive and Autonomic Considerations

Cognitive decline may affect medication adherence, perception of thirst, recognition of genitourinary symptoms, and the ability to apply sick-day rules, thereby increasing the risk of dehydration and preventable adverse events. Therefore, cognitive status, medication self-management, caregiver availability, and the capacity to follow written instructions should be considered before treatment initiation. Emerging evidence links SGLT2 inhibition to reduced cerebral oxidative stress and improved neurovascular coupling [45]. However, these findings remain preliminary and should not be used as a primary rationale for treatment initiation in cognitively impaired older adults.

#### 3.6.4. Falls and Orthostatic Tolerance

Falls represent a major concern in geriatric pharmacotherapy because they are associated with fractures, loss of independence, institutionalisation, and mortality. Although SGLT2 inhibitors generally induce only modest reductions in blood pressure, the risk of falls should still be considered in older adults with orthostatic hypotension, frailty, polypharmacy, gait instability, recurrent falls, autonomic dysfunction, low baseline systolic blood pressure, or concomitant diuretic therapy. Nevertheless, careful review of concomitant antihypertensive treatment, attention to hydration, and monitoring for dizziness or postural symptoms are essential to minimise the risk of falls [46].

#### 3.6.5. Individualisation and Shared Decision-Making

Therapeutic choices in advanced age should integrate life expectancy, functional status, cognitive function, goals of care, treatment burden, caregiver support, and patient preferences. For a robust 80-year-old with T2DM, CKD, or HFpEF, SGLT2 inhibitors may offer a favourable benefit–to–risk ratio. Conversely, in a bed-bound nonagenarian with severe frailty, advanced dementia, poor oral intake, recurrent dehydration, repeated falls, or limited life expectancy, treatment initiation may be inappropriate or may reasonably be deferred. Temporary withholding should also be considered during acute illness, prolonged fasting, vomiting, diarrhoea, severe dehydration, active genitourinary infection, or perioperative periods. Multidisciplinary evaluation involving cardiologists, geriatricians, diabetologists, nephrologists, pharmacists, nurses, and caregivers may optimise patient selection, monitoring, adherence, and timely treatment interruption when clinically appropriate [47].

### 3.7. Practical Implementation in Geriatric Care

#### 3.7.1. Candidate Selection

SGLT2 inhibitors should be considered in older adults with T2DM, HF, or CKD when an approved indication is present and renal eligibility is compatible with the specific agent. However, in geriatric practice, treatment eligibility should not be based on eGFR alone, but should integrate frailty status, functional capacity, volume status, nutritional reserve, cognitive function, concomitant diuretic therapy, infection risk, life expectancy, and patient-centred goals of care [48]. Particular caution is required in vulnerable subgroups that are underrepresented in clinical trials but frequently encountered in geriatric practice. These include nursing home residents or homebound patients, individuals with recurrent dehydration or poor oral intake, severe orthostatic hypotension, advanced frailty, active diabetic foot disease, recurrent fungal or genitourinary infections, advanced dementia, and those receiving end-of-life or comfort-focused care. In these settings, the benefit–risk balance may be less favourable, and treatment decisions should be individualised, prioritising symptom control, treatment burden, and patient-centred goals of care rather than long-term cardiometabolic prevention.

**Figure 5:**
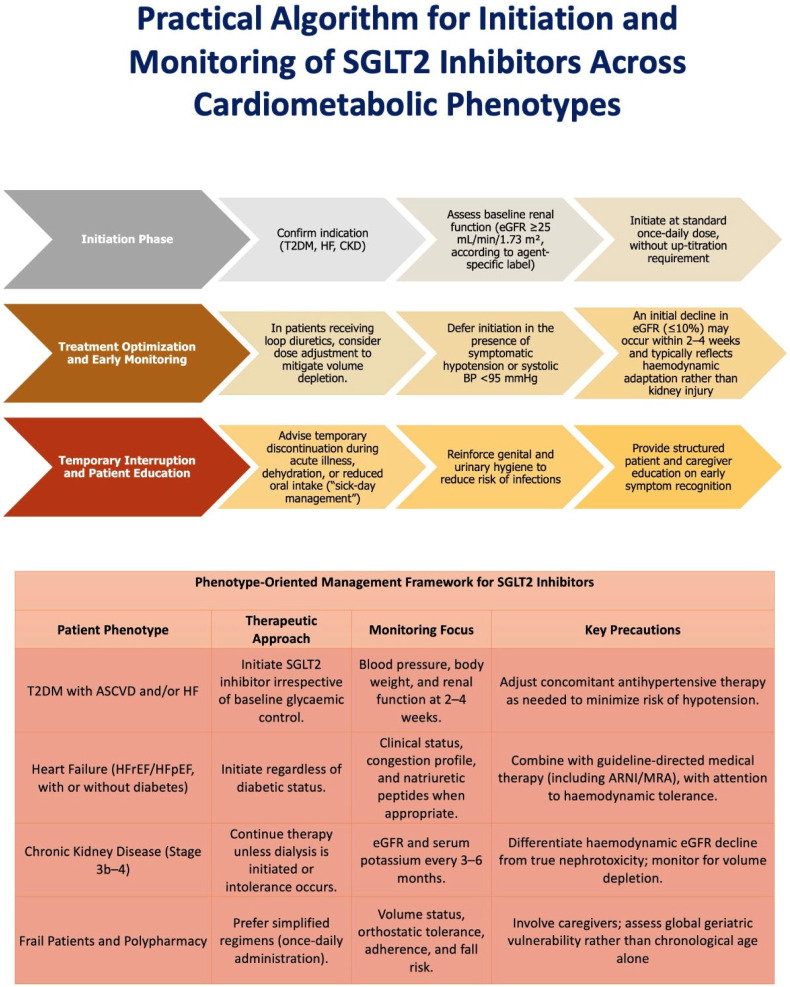
Practical algorithm for the initiation and monitoring of SGLT2 inhibitors across cardiometabolic phenotypes. This figure illustrates a pragmatic framework for the safe initiation and clinical management of SGLT2i inhibitors in patients with cardiometabolic diseases. The algorithm is structured into three sequential phases. In the “Select and Start” phase, clinicians identify appropriate candidates, including patients with T2DM, HF, or CKD, verify renal eligibility according to the approved indication and the specific SGLT2 inhibitor, while also assessing volume status, frailty, nutritional reserve, and concomitant diuretic therapy. The “Pause and Adjust” phase highlights situations requiring caution or dose adjustment, such as concomitant loop diuretic therapy, low systolic blood pressure, or the expected transient decline in estimated glomerular filtration rate during the first weeks of treatment. The “Stop and Educate” phase emphasises temporary treatment interruption during acute illness or severe dehydration, as well as the importance of patient education, including sick-day rules and genital hygiene. The lower matrix integrates these principles into a phenotype-oriented clinical guide, outlining suggested therapeutic strategies, monitoring priorities, and key precautions for patients with CKD and CVD, HF with or without T2DM, CKD, and frailty or polypharmacy.

The most suitable candidates are those with recent HF hospitalisation, progressive renal decline, or sub-optimal diuretic control. Age alone should not determine treatment eligibility; instead, biological age, frailty status, functional capacity, cognitive profile, comorbidity burden, and treatment goals should guide patient selection. A simplified clinical algorithm may facilitate the safe initiation and monitoring of SGLT2i across different cardiometabolic phenotypes, particularly in complex or frail patients (Figure 5). Practical recommendations for SGLT2i use in older patients across different clinical settings are summarised in Table 4.

#### 3.7.2. Initiation Strategy

Therapy can be initiated at standard doses (empagliflozin 10 mg, dapagliflozin 10 mg, canagliflozin 100 mg, ertugliflozin 5 mg) once daily, preferably in the morning. Hydration status and renal function should be verified before starting. In patients already receiving loop or thiazide diuretics, concomitant diuretic therapy should be reassessed before initiation. Dose adjustment may be appropriate in those with low blood pressure, orthostatic symptoms, euvolaemia or hypovolaemia, poor oral intake, or previous dehydration, but should be individualised rather than applied systematically.

#### 3.7.3. Monitoring and Follow-Up

Clinical review after 2–4 weeks should include BP, weight, orthostatic symptoms, and serum creatinine. A transient GFR fall of ≤10% is acceptable and typically stabilises. In elderly patients with HFpEF, autonomic dysfunction, CKD stage 3–4, concomitant loop diuretics, low baseline systolic blood pressure, or recurrent falls, earlier reassessment may be appropriate. Monitoring should include sitting and standing blood pressure when feasible, body weight, hydration status, symptoms of dizziness or weakness, renal function, electrolytes, and signs of persistent congestion or excessive decongestion. Laboratory checks every 3–6 months thereafter are adequate. Education on genital hygiene and “sick-day” management is crucial, especially for cognitively impaired patients. Telephone or telehealth follow-up can ensure adherence and early recognition of side effects. In patients with CKD stage 4, advanced age, frailty, concomitant diuretic therapy, or borderline volume status, earlier clinical and biochemical reassessment may be appropriate. Monitoring should include renal function, electrolytes, blood pressure (including orthostatic measurements when feasible), body weight, hydration status, symptoms of dizziness or weakness, and intercurrent illness. Particular caution is required in patients with HFpEF, autonomic dysfunction, or limited preload reserve, in whom even modest reductions in intravascular volume may lead to symptomatic hypotension or functional decline. Closer monitoring or avoidance of treatment initiation may be appropriate in patients with recurrent dehydration, unstable clinical conditions, poor adherence, or limited ability to recognise or report early symptoms of adverse events.

#### 3.7.4. Practical Integration with Heart Failure Therapy in Older Adults

In older patients with HF, SGLT2 inhibitors should be integrated into guideline-directed therapy according to congestion status, blood pressure tolerance, renal function, frailty, and concomitant diuretic use. Their mild natriuretic and osmotic diuretic effect may be clinically useful in congestion-prone patients, but it may also increase the risk of volume depletion when combined with loop diuretics, particularly in frail individuals, patients with poor oral intake, or those with low baseline systolic blood pressure. Therefore, loop diuretic dose should not be automatically reduced in all patients, but reassessed individually after treatment initiation according to weight change, congestion, orthostatic symptoms, renal function, and blood pressure.

Particular caution is required in older patients with HFpEF, stiff ventricles, autonomic dysfunction, or limited preload reserve, in whom even modest intravascular volume reduction may precipitate dizziness, hypotension, fatigue, worsening renal function, or functional decline. In these patients, early reassessment within 1–2 weeks may be preferable to routine follow-up at 4 weeks.

When SGLT2 inhibitors are introduced together with ARNI, beta-blockers, or mineralocorticoid receptor antagonists, treatment sequencing should be individualised. In patients with adequate blood pressure and persistent congestion, early initiation may be appropriate. In contrast, in patients with borderline blood pressure, recurrent falls, orthostatic intolerance, advanced frailty, or recent acute illness, a stepwise approach with close monitoring may be safer. Blood pressure tolerance should be assessed using both seated and standing measurements whenever feasible, especially in patients with autonomic dysfunction or polypharmacy. In highly vulnerable patients, including those with advanced frailty or severe orthostatic intolerance, a more conservative approach with delayed initiation or treatment avoidance may be appropriate.

#### 3.7.5. Integration with Multidisciplinary Programmes

This multidisciplinary approach is particularly important when SGLT2 inhibitors are introduced in patients already receiving loop diuretics, ARNI, beta-blockers, or MRA, because treatment sequencing and dose adjustments should be guided by blood pressure tolerance, renal function, potassium levels, congestion status, frailty, and risk of orthostatic hypotension. Embedding SGLT2i initiation within heart-failure or CKD clinics facilitates rapid titration of companion drugs (ARNI, MRA, SGLT2i) and provides a structured environment for elderly follow-up [37]. Nurses and pharmacists can reinforce education, monitor weight trends, and adjust therapy remotely.an approach proven to lower readmission rates. Blood pressure tolerance may be reduced in elderly patients with autonomic dysfunction, necessitating gradual treatment introduction and close monitoring of orthostatic symptoms.

#### 3.7.6. Health-Economic and Ethical Perspectives

The reduction in recurrent HF admissions and dialysis initiation confers substantial cost savings that offset the drug price within 12 months. For health systems under budget constraints, prioritising high-risk elderly patients maximises cost-effectiveness. From an ethical perspective, treatment decisions should aim to avoid ageism while also respecting frailty, prognosis, patient preferences, treatment burden, and the goals of care. Decisions should respect patient autonomy, balancing longevity with comfort and quality of life.

## 4. Discussion

This review highlights the evolving role of SGLT2 inhibitors as a cornerstone therapy across the cardiorenal continuum, with particular relevance in older adults, in whom HF, CKD, and T2DM frequently coexist.

A key finding from the available evidence is that the CV effects of SGLT2 inhibitors are heterogeneous across endpoints. The most consistent and robust benefit is the reduction in HHF, observed across a wide range of clinical settings, including patients with and without T2DM and across the spectrum of ejection fraction. In contrast, effects on CV death and major adverse CV events are more variable and appear to depend on baseline CV risk, population characteristics, and trial design. This distinction is particularly relevant in older adults, in whom therapeutic expectations should be aligned with the predominant benefit on HF outcomes rather than assuming a uniform reduction in atherosclerotic events.

Another important aspect concerns the translation of clinical trial evidence into geriatric practice. Although subgroup analyses consistently suggest preserved relative efficacy in older age groups, most available data remain age-stratified rather than truly geriatric. Patients with advanced frailty, cognitive impairment, severe dependency, or those residing in nursing homes are largely underrepresented in randomised trials. As a result, the generalisability of trial findings to the most vulnerable populations remains uncertain, and clinical decision-making should rely on a more comprehensive geriatric assessment rather than chronological age alone.

From a mechanistic perspective, SGLT2 inhibitors exert a range of hemodynamic, metabolic, and renal effects that are biologically plausible in counteracting key pathways of cardiometabolic ageing, including congestion, oxidative stress, and endothelial dysfunction. However, it is important to distinguish between mechanistic hypotheses and outcomes that have been directly demonstrated in clinical trials. While these mechanisms provide a coherent pathophysiological framework, their relative contribution to clinical benefit, particularly in frail or very elderly patients, remains incompletely defined.

Safety and tolerability represent critical considerations in older populations. Overall, SGLT2 inhibitors have a favourable safety profile, with low intrinsic risk of hypoglycaemia. However, specific vulnerabilities must be taken into account, including the risk of volume depletion, orthostatic hypotension, genitourinary infections, and, in selected conditions, euglycemic ketoacidosis. These risks are particularly relevant in frail individuals, patients with poor oral intake, polypharmacy, or limited self-care capacity, and require careful patient selection, monitoring, and education.

Importantly, the benefit–risk profile of SGLT2 inhibitors is not uniform across all older adults. Robust individuals with preserved functional status may derive substantial benefit, similar to that observed in clinical trials, whereas in frail, undernourished, or highly dependent patients, tolerability, treatment burden, and competing risks may attenuate the net clinical benefit. In this context, a phenotype-oriented and patient-centred approach is essential, integrating frailty status, comorbidities, life expectancy, and individual goals of care.

Finally, implementation in clinical practice requires integration within multidisciplinary care pathways, particularly in complex patients with HF and CKD. Early identification of suitable candidates, appropriate adjustment of concomitant therapies, and structured follow-up are key elements to maximise benefit and minimise adverse events.

This review has several limitations. Although a structured approach to literature identification was adopted, including PRISMA-based selection, the synthesis remains narrative and therefore subject to potential selection bias. In addition, the heterogeneity of study populations, endpoints, and trial designs limits direct comparisons across studies, particularly in relation to absolute risk reduction and number needed to treat. Finally, evidence in very old, frail, or institutionalised populations remains limited and is largely derived from subgroup or observational analyses.

Overall, the available evidence supports the use of SGLT2 inhibitors as an effective and generally well-tolerated therapeutic option in older adults with cardiometabolic disease. However, their use should be individualised, with careful consideration of frailty, clinical context, and patient-centred goals.

## 5. Conclusions

SGLT2 inhibitors have transformed the management of cardio–renal–metabolic disease by reducing HHF, slowing renal decline, and improving outcomes across a broad spectrum of patients. However, these benefits should be interpreted in the context of the specific endpoint and clinical context. A reduction in HHF represents the most consistent CV effect, whereas effects on CV death and MACE are more heterogeneous across trials and populations. In older adults, these benefits are particularly relevant, given the frequent coexistence of HF, CKD, T2DM, multimorbidity, and polypharmacy. Their once-daily oral administration, low intrinsic risk of hypoglycaemia, and generally favourable tolerability profile make SGLT2 inhibitors an attractive therapeutic option in appropriately selected elderly patients.

Nevertheless, evidence in very old, frail, underweight, cognitively impaired, dependent, or nursing home populations remains limited. Much of the available data in these groups derives from subgroup analyses, post hoc evaluations, meta-analyses, or observational studies rather than dedicated randomised trials. Accordingly, established clinical benefits, such as reductions in HHF and progression of kidney disease, should be clearly distinguished from mechanistic hypotheses related to inflammation, oxidative stress, mitochondrial function, endothelial dysfunction, and biological ageing. Implementation in geriatric practice requires a comprehensive and individualized approach that integrates frailty status, functional capacity, nutritional reserve, renal function, orthostatic tolerance, hydration status, cognitive function, medication self-management, caregiver support, and life expectancy. In robust or pre-frail older adults with appropriate indications, SGLT2 inhibitors may offer a favourable benefit–risk profile. Conversely, in patients with advanced frailty, poor oral intake, recurrent dehydration, repeated falls, severe cognitive impairment, or limited prognosis, treatment initiation may be inappropriate or should be deferred.

Overall, SGLT2 inhibitors should be incorporated into geriatric care pathways through a balanced, patient-centred strategy that weighs established cardiorenal benefits against individual vulnerability, treatment burden, and goals of care. Thus, in older adults, SGLT2 inhibitors should be presented not as a uniform CV protection strategy, but as endpoint-specific therapies with particularly robust evidence for reducing HF events and renal progression, requiring individualized integration into complex geriatric care.

## Figures and Tables

**Table 1:** Mechanistic spectrum of SGLT2 inhibition relevant to the elderly.

Domain	Mechanism	Expected Clinical Impact
**Hemodynamic**	Osmotic diuresis, natriuresis, reduced preload/afterload	Fewer HF exacerbations, lower BP variability
**Metabolic**	Mild glycosuria, weight & uric-acid reduction, ketone shift	Improved energetics, lower inflammation
**Renal**	Restored tubuloglomerular feedback, ↓ intraglomerular pressure	Slower eGFR decline, less albuminuria
**Endothelial**	↓ oxidative stress, improved NO availability	Better arterial compliance, microvascular flow
**Cardiac cellular**	↓ Na^+^/H^+^ exchanger activity, ↓ Ca^2+^ overload	Enhanced diastolic function, anti-remodelling

Abbr.: SGLT2: sodium–glucose co-transporter 2; HF: heart failure; BP: blood pressure; eGFR: estimated glomerular filtration rate; NO: nitric oxide; Na^+^: sodium; H^+^: hydrogen ion; Ca^2+^: calcium. The symbol “↓” indicates a reduction/decrease.

**Table 4:** Practical use of SGLT2 inhibitors in older patients.

Clinical Setting	Suggested Approach	Monitoring	Key Precautions
**T2DM with ASCVD and/or HF**	Initiate at standard dose when indication is present	BP, body weight, renal function at 2–4 weeks	Reassess concomitant antihypertensive and diuretic therapy to avoid hypotension
**HFrEF (with or without diabetes)**	Initiate regardless of glycemic status as part of GDMT	Clinical status, congestion, renal function	Combine with ARNI, beta-blockers, and MRA; monitor BP tolerance and volume status
**HFpEF (especially elderly phenotype)**	Consider initiation after evaluation of volume status and BP reserve	Sitting and standing BP, symptoms, renal function (early reassessment at 1–2 weeks if frail)	Risk of hypotension in patients with stiff ventricles, autonomic dysfunction, or limited preload reserve; avoid excessive decongestion
**CKD (stage 3b–4)**	Continue/initiate according to indication and renal eligibility	eGFR, potassium every 3–6 months	Distinguish hemodynamic eGFR dip from true nephrotoxicity; reassess during acute illness or dehydration
**Frail patients/polypharmacy**	Individualised approach; simplify regimen where possible	Adherence, hydration status, orthostatic symptoms, weight	Avoid initiation in active dehydration, poor oral intake, or severe frailty; involve caregivers
**Patients on loop diuretics**	Do not systematically reduce dose; reassess individually after initiation	Weight, congestion, BP, renal function within 1–2 weeks	Increased risk of volume depletion; adjust diuretics based on clinical response
**Autonomic dysfunction/orthostatic intolerance**	Initiate cautiously with close follow-up	Sitting and standing BP, dizziness, falls	Increased risk of symptomatic hypotension; consider slower titration and earlier review

**Abbreviations:** SGLT2: sodium–glucose co-transporter 2; T2DM: type 2 diabetes mellitus; ASCVD: atherosclerotic cardiovascular disease; HF: heart failure; HFpEF: heart failure with preserved ejection fraction; HFrEF: heart failure with reduced ejection fraction; DM: diabetes mellitus; CKD: chronic kidney disease; eGFR: estimated glomerular filtration rate; BP, blood pressure; ARNI: angiotensin receptor–neprilysin inhibitor; MRA, mineralocorticoid receptor antagonist.

## Data Availability

No new data were created or analyzed in this study.
